# Precipitation and soil nutrients determine the spatial variability of grassland productivity at large scales in China

**DOI:** 10.3389/fpls.2022.996313

**Published:** 2022-09-09

**Authors:** Xianxian Wang, Ru Wang, Jie Gao

**Affiliations:** ^1^College of Life Sciences, Xinjiang Normal University, Urumqi, China; ^2^Institute of Ecology and Key Laboratory of Earth Surface Processes of Ministry of Education, College of Urban and Environmental Sciences, Peking University, Beijing, China

**Keywords:** grassland productivity, leaf traits, climate change, soil nutrients, desert steppe

## Abstract

Changes in net primary productivity (NPP) to global change have been studied, yet the relative impacts of global change on grassland productivity at large scales remain poorly understood. Using 182 grassland samples established in 17 alpine meadows (AM) and 21 desert steppes (DS) in China, we show that NPP of AM was significantly higher than that of DS. NPP increased significantly with increasing leaf nitrogen content (LN) and leaf phosphorus content (LP) but decreased significantly with increasing leaf dry matter content (LDMC). Among all abiotic factors, soil nutrient factor was the dominant factor affecting the variation of NPP of AM, while the NPP of DS was mainly influenced by the changing of precipitation. All abiotic factors accounted for 62.4% of the spatial variation in the NPP of AM, which was higher than the ability to explain the spatial variation in the NPP of DS (43.5%). Leaf traits together with soil nutrients and climatic factors determined the changes of the grassland productivity, but the relative contributions varied somewhat among different grassland types. We quantified the effects of biotic and abiotic factors on grassland NPP, and provided theoretical guidance for predicting the impacts of global change on the NPP of grasslands.

## Introduction

Grassland accounts for about a quarter of the global land area and is an important component of the terrestrial ecosystem (Pecina et al., 2019). Grasslands can not only effectively contributes to the global carbon cycle, but also plays a key role in regulating climate and soil conservation (Wang et al., 2019; Zhang et al., 2020). Grassland ecosystems are monospecific and structurally simple, making them more climate-sensitive than forest ecosystems (Liang and Gornish, 2019). Different types of grasslands (such as meadow steppe, typical steppe, desert steppe, and alpine steppe) also have obvious differences in ecosystem functions. Therefore, it is of great significance for us to investigate how environmental factors affect the functions of various grasslands in the context of global change (Anderegg et al., 2015; Seddon et al., 2016).

Net primary productivity (NPP) reflects the amount of carbon fixed by photosynthesis in a certain ecosystem per unit of time and space (Gang et al., 2018). NPP represents the energy that plants can use for growth, development, and reproduction, and is also the material basis for the survival and reproduction of biological groups (Sun and Du, 2017). Numerous studies have found that biological and abiotic factors jointly determine the temporal and spatial changes of NPP in grassland ecosystems (Shi et al., 2022). For example, Henry et al. (2018) found that precipitation controlled the spatio-temporal variation of grassland NPP, while Gong H. et al. (2020) believed that the availability of soil N and P content were the key factors that shaping the distribution pattern of grassland NPP. In recent years, with the development of functional trait research, leaf traits were widely used to predict the changes of NPP (Forrestel et al., 2017; Li et al., 2020; Schrader et al., 2021; Jiao et al., 2022). There remains no consensus on the dominant factors of spatial-temporal variation in grassland NPP (Henry et al., 2018; Wei et al., 2020).

Alpine meadows (AM) are mainly distributed in the alpine plateau region, which located at higher altitude regions. While desert steppes (DS) are mainly located in temperate regions with little mean annual precipitation (MAP) and are mainly distributed in northwest China (Zhou et al., 2020). Environmental factors driving the NPP of various grasslands vary considerably due to the difference in hydrothermal conditions and the altitude at which they are located (Sun et al., 2021). Drought stress significantly affects the photosynthesis and metabolism of the plants in DS (Grilli et al., 2017), therefore, precipitation is a key limiting factor of the NPP in DS. With the decline in MAP, the NPP of the DS decreased significantly (Chen et al., 2022). However, many studies have found that rainfall does not directly affect plant metabolism, but further influences plant uptake of soil nutrients by acting on soil microbial respiration and accelerating soil nutrient leaching and transformation (Gao et al., 2021). Soil is the direct living environment for plants. Plants absorb water and nutrients from the soil for photosynthesis. Soil nutrients control the photosynthesis of grass plants and affect the NPP of grasslands (Wieder et al., 2015). Soil N content can mitigate the effects on desert grassland NPP due to reduced precipitation, while soil P content is an essential element for plant energy transfer (Baldarelli et al., 2021), and soil pH represents soil fertility, which is a key soil factor to maintain NPP in desert grassland ecosystem (Zhang et al., 2021). Plants adapt to climate change by altering their traits (Wang et al., 2017b). Plants adapt to drought stress by reducing the leaf dry matter content (LDMC) and increased the leaf nitrogen content (LNC; Mahaut et al., 2020). Plants with lower specific leaf area (SLA) had a stronger ability to retain water and generally have a longer life span (Gong and Gao, 2019; Luo et al., 2020).

Compared with the DS, the structure and function of the AM are more sensitive to climate change (Pries et al., 2017). Zhang et al. (2019) found that light intensity significantly affects the NPP of the AM through the brightening experiment. NPP of the AM is also limited by low temperature and has no significant relationship with precipitation (Pan et al., 2021). Leaf traits represent the adaptability of alpine grassland function to low temperature, and plants with smaller leaf area (LA) are better able to avoid frost and heat damage (Dong et al., 2017). LN and LP are essential for plant growth and development (Heineman et al., 2016), and grassland NPP largely depends on plant availability to nitrogen and phosphorus (Wieder et al., 2015). LDMC is also closely related to grassland NPP, and Xu et al. (2018) found that grassland NPP is positively correlated with LDMC. The spatio-temporal variation of NPP of different grassland types is mainly influenced by a combination of environmental and biological factors, but there remains no consensus on the relative contribution of these factors (Gong Y. H. et al., 2020; Jochum et al., 2020; Shi et al., 2020).

Based on the field data from 182 grasslands in 17 AM and 21 DS in China, we sought to identify the main drivers of NPP in different grassland ecosystems. To answer this question, we proposed the following hypotheses: (1) The NPP of AM is significantly higher than DS. (2) Precipitation is the driving factor of NPP of DS, while NPP of AM is the combined effect of precipitation and temperature. (3) Climate factors are the key environmental factors dominating NPP of AM and that of DS, however, soil nutrient factors also play a non-negligible role.

## Materials and methods

### Study area and plot description

**FIGURE 1:**
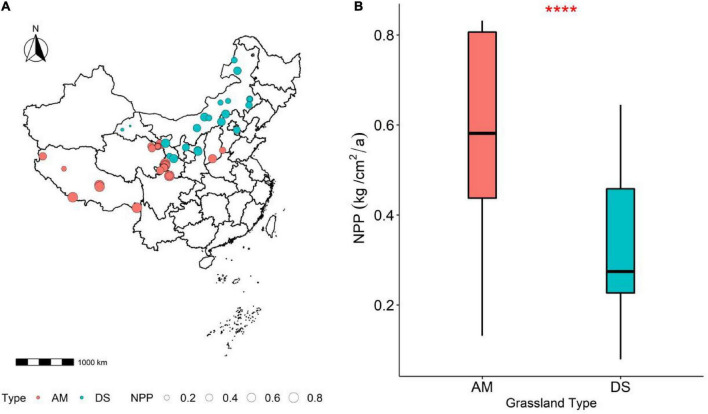
Comparison of the variability of different grassland NPP. **(A)** Geographical location of the grassland plots in this study; **(B)** variability of the NPP of AM and DS. AM represents alpine meadows and DS represents desert steppes. ****Represents *P* < 0.0001.

China has a vast territory and a diverse climate, with possesses many types of grasslands. These grasslands are mainly distributed in arid and semi-arid areas in northern China and the Qinghai-Tibet Plateau (Yu et al., 2021; Shen et al., 2022). The large spatial scale and the richness of climate types provide conditions for exploring the spatio-temporal variation of NPP of grasslands (Huang et al., 2021). The study used data from 182 grasslands in 17 AM in the southwest region and 21 DS in the north central region in China (Figure 1A). Our study sites range in latitude from 19.1° to 53.5° north and longitude from 79.72° to 121.1° east, with elevations ranging from 13 to 5,000 m.

### Community leaf traits data

Leaf traits are widely used to detect the adaptability of plants to the living environment (Hikosaka et al., 2021). The traits we selected included LA, SLA, LDMC, LN, LP, and leaf N/P ratio. These traits are closely related to light interception, resource utilization, growth, and development of plants (Brun et al., 2022). Previous studies at leaf traits usually focused on the species level (Butler et al., 2017). However, the effects of competitive asymmetry (i.e., the magnitude and consequences of competition affecting each party) related to interspecies functional characteristics has been neglected (Vázquez et al., 2007). We used community weighted average trait (CWM_*i*_) to represent average trait values in grassland.


(1) $$\text{CWM}=\sum_{\text{i}=1}^{\text{S}}\text{Di}\times \text{Traiti}$$

where CWM_*i*_ represents the weighted trait value of the community, and D_i_ represents the abundance of the target species.

### Environmental factors and net primary productivity data

Numerous studies have found that grassland NPP is influenced by climate factors, especially mean annual temperature (MAT) and MAP (Sloat et al., 2018). MAT, mean coldest monthly temperature (MCMT), mean warmest monthly temperature (MWMT), and MAP were extracted from the WorldClim global climate layer at a spatial resolution of 1 km (Conradi et al., 2020). Light is a key climatic factor affecting photosynthesis (Kramer, 1981). We hypothesized that annual sunshine hours (ASD) may be a key predictor of NPP. ASD and mean annual evaporation (MAE) were also obtained from the Meteorological Data Center of the China Meteorological Administration.^1^

We extracted from 250 m resolution of the grid in the top 30 cm soil layer soil pH, soil nitrogen,^2^ and the content of soil effective phosphorus.^3^

NPP data from the national aeronautics and space administration (NASA^4^), the site offers from 2000 to 2015, a resolution of 250 m by 250 m of NPP. This dataset was derived from the widely used Medium Resolution Imaging Spectroradiometer (MOD13Q1) product, calculated using the C5 MOD17 algorithm, and verified by data from flflux towers (Li et al., 2020).

### Data analysis

We used a significant difference test at the 0.05 significance level to test whether there was a significant difference between NPP in AM and DS (Figure 1). We also tested CWM_*i*_ differences between different grassland types at α = 0.05 (Chen et al., 2020; Supplementary Figure 1). Significant difference tests were performed using the R package agricolae (version 4.1.0, R Core Team, 2020).

We tested the effects of environmental factors and community functional traits on NPP using general linearity. *R*^2^ represents the goodness of fit of the model. Linear regression was performed using the R package lme4 (R Core Team, 2020).

**FIGURE 2:**
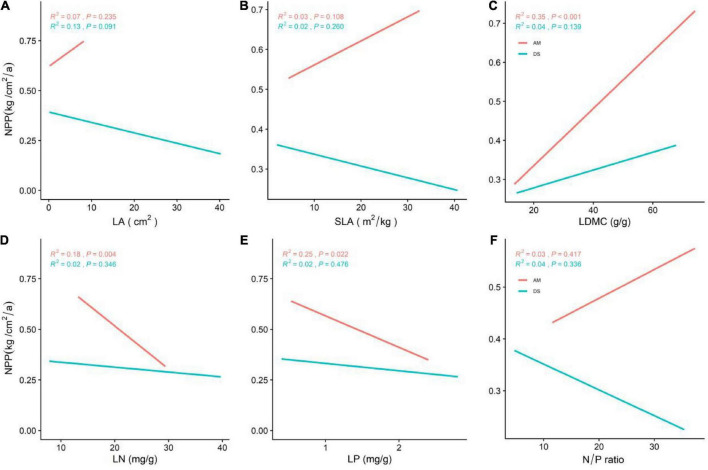
General linear correlations between community leaf traits and the NPP of AM and DS. **(A)** General linear relationship between LA and the NPP of AM and DS; **(B)** general linear relationship between SLA and the NPP of AM and DS; **(C)** general linear relationship between LDMC and the NPP of AM and DS; **(D)** general linear relationship between LN and the NPP of AM and DS; **(E)** general linear relationship between LP and the NPP of AM and DS; **(F)** general linear relationship between N/P and the NPP of AM and DS. LA represents leaf area; SLA represents specific leaf area; LDMC represents leaf dry matter content; LN represents leaf nitrogen content; LP represents leaf phosphorus content; N/P represents leaf nitrogen and phosphorus ratio. The red line represents the NPP of AM and the green line represents the NPP of DS; *R*^2^ represents the fit of the model, and *P* represents the correlation.

Our preliminary results indicate that most functional traits have no significant linear relationship with NPP (Figure 2). Therefore, in subsequent studies, we mainly explore the impact of environmental factors on NPP.

We used a generalized additive model (GAM) to test the effects of climate and soil factors on NPP. This model is composed of parametric variables and non-parametric variables (Zou et al., 2016). Non-metric multidimensional scaling analysis (NMDS) was used to reflect the fit of GAM (Zou et al., 2016).


(2) $$\text{g}\,\left[E\,\left(Y|X\right)\right]=\sum_{i}\beta \,i\,X\,i+\sum_{j}f\,i\,\left(X\,i\right)+\varepsilon$$

where g(●) denotes the connection function, the form of which depends on the specific form and can be interpreted as the variable Y distribution. *E* is a random error term that can be interpreted as a normally distributed function named constant variable connection, and the connection function takes the form g(u) = u, u = E(Y | X), E(*E*| X) = 0. X_i_ is the explanatory variable that strictly follows the parametric form of the explanatory variables, βi is the corresponding parameter, and f_*j*_(●) is the smoothing function corresponding to the explanatory variable X_j_ that follows a non-parametric form. In our study, the spline smoothing function S(●) is chosen to fit, thin-slab spline smoothing is chosen to fit the function between different nodes, and least squares is used to estimate each smoothing function S(●).

## Results

### Effects of leaf traits on net primary productivity of grasslands

The NPP of AM was significantly higher than that of DS (*P* < 0.001), and most functional traits of AM and DS were also significantly different (Figure 1B). Except that the N/P of AM was lower than that of DS, other leaf traits were significantly higher than that of DS (Supplementary Figure 1). The NPP of different grassland types increased significantly with the increase of LDMC (Figure 2C), and decreased significantly with the increase of LN and LP (Figures 2D,E). NPP of AM and DS showed opposite trends with the increase of LA, SLA, and leaf N/P ratio (Supplementary Figures 1A,B). With the increase of the above leaf traits, NPP increased significantly of AM, while NPP decreased significantly of DS. Among all leaf traits, LDMC had better predictive power for NPP of AM (*R*^2^ = 0.35, *P* < 0.001; Figure 2C) and LA had better predictive power for NPP of DS (*R*^2^ = 0.13, *P* < 0.001; Figure 2A).

### Effects of climate and soil nutrients on net primary productivity of grasslands

**FIGURE 3:**
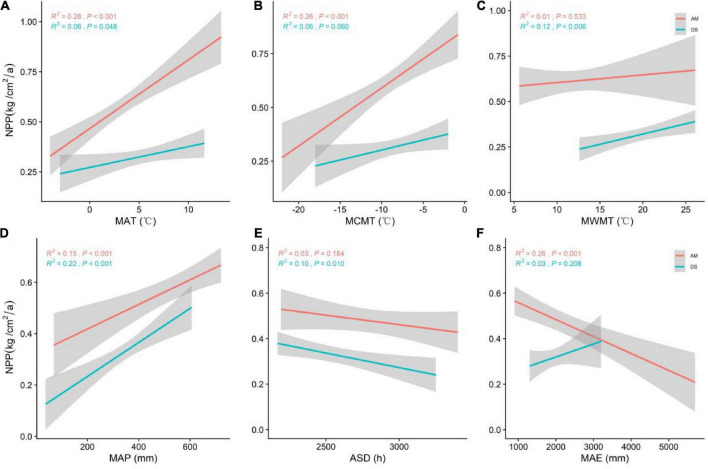
General linear regression analysis between climate factors and the NPP of AM and DS. **(A)** General linear relationship between MAT and the NPP of AM and DS; **(B)** general linear relationship between MCMT and the NPP of AM and DS; **(C)** general linear relationship between MWMT and the NPP of AM and DS; **(D)** general linear relationship between MAP and the NPP of AM and DS; **(E)** general linear relationship between ASD and the NPP of AM and DS; **(F)** general linear relationship between MAE and the NPP of AM and DS. MAT represents mean annual temperature; MCMT represents mean coldest monthly temperature; MWMT represents mean warmest monthly temperature; MAP represents mean annual precipitation; ASD represents annual sunshine hours; MAE represents mean annual evaporation. The shaded area shows a 95% confidence interval. The red line represents the NPP of AM, and the green line represents the NPP of DS; *R*^2^ represents the model fit, and *P* represents the correlation.

The NPP of AM was significantly positively correlated with MAT and MCMT and negatively correlated with MAE. The NPP of DS was significantly positively correlated with both MWMT and MAP (Figure 3). MAT (*R*^2^ = 0.28, *P* < 0.001) was the best climate factor in predicting the change of NPP of AM. MAP (*R*^2^ = 0.22, *P* < 0.001) was the best climate factor in predicting the change of NPP of DS.

**FIGURE 4:**
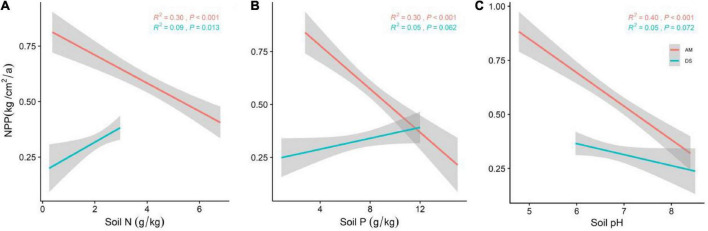
General linear regression analysis between soil nutrient factors and the NPP (soil N, soil P and soil PH) of AM and DS. **(A)** General linear relationship between soil N and the NPP of AM and DS; **(B)** general linear relationship between soil P and the NPP of AM and DS; **(C)** general linear relationship between soil pH and the NPP of AM and DS. The shaded area shows a 95% confidence interval. The red line represents the NPP of AM and the green line represents the NPP of DS; *R*^2^ represents the model fit and *P* represents the correlation.

We further analyzed the effects of soil nutrient factors on the NPP of different types of grasslands (Figure 4). The prediction ability of soil N, soil P, and soil pH on NPP of AM were 0.30, 0.30, and 0.40, respectively. Among all soil nutrient factors, except the positive correlation between soil N and the NPP of DS, the other factors have no significant effect (*P* > 0.05).

### Soil factors dominate the variation of grassland net primary productivity

**FIGURE 5:**
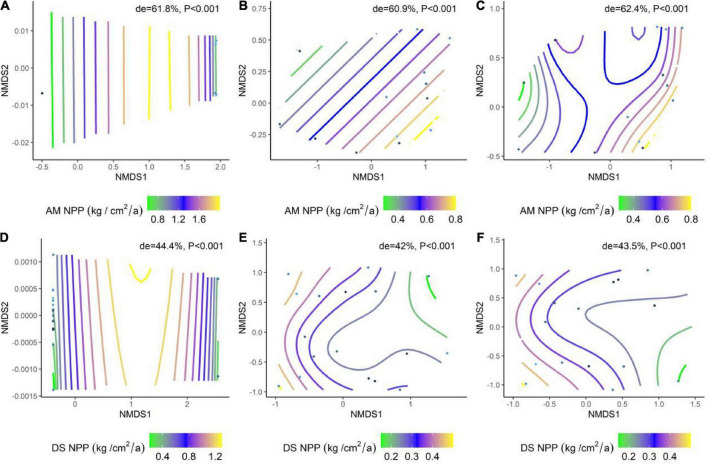
The results of the generalized additive model (GAM) fitted the effects of environmental factors and climate factors on grassland NPP. **(A)** NMDS ranking of soil factors and the NPP of AM; **(B)** NMDS ranking of climate factors and the NPP of AM; **(C)** NMDS ranking of environmental factors and the NPP of AM; **(D)** NMDS ranking of soil factors and the NPP of DS; **(E)** NMDS ranking of climate factors and the NPP of DS; **(F)** NMDS ranking of environmental factors with the NPP of DS. The color spline shows the fit of trait data from high (green) to low (yellow) values during the ranking. Trait overlays indicate that abiotic factors represented by points on the NMD are associated with higher or lower trait values, consistent with the colored trait gradient. Note that if the relationship between NPP and abiotic factors is linear, the gradient spline lines will be parallel. non-linear relationships between NPP and abiotic factors are represented by curve splines; de indicates the deviation explained by the corresponding model.

We analyzed the effects of abiotic factors on the NPP of AM and DS based on a generalized additive model with non-metric multidimensional scaling (NMDS) ordering. All environmental factors jointly explained 62.4% of NPP in AM and 43.5% of NPP in DS (Figures 5C,F). Among them, the soil factor explained the variation of NPP in different types of grasslands to a greater extent than the climate factor (Figures 5A,B,D,E). Soil factors are the dominant factors driving the spatial and temporal variation of NPP in grasslands, and climate factors also play an important role.

## Discussion

There were significant spatial differences in NPP of different grassland types, and the NPP of AM was significantly higher than that of DS. Significant differences in NPP were closely related to habitat heterogeneity (Hikosaka et al., 2021). Due to the differences in biological or abiotic factors in different regions, there are significant differences in NPP of grassland in different regions (Li et al., 2019). AM are mainly distributed in the cold and high-altitude area of southwest China, with low temperature, strong radiation, thin soil layer, long soil freezing period, and neutral soil reaction. And DS are mainly distributed in the arid and semi-arid areas in the middle and north of China, which is characterized by lack of rainfall, strong evaporation, loose soil, lack of water, and low organic matter content. Therefore, differences in local hydrothermal conditions and community structure may be important reasons for the significant differences in NPP between AM and DS.

A large number of studies have found that the community leaf traits are closely related to the NPP of grasslands (Wang et al., 2017b). Our results found that the NPP of different grasslands increased significantly with the decreased of LN and LP, and increased significantly with the increase of LDMC. Nitrogen is the main component of chlorophyll, which is used to accumulate organic matter through light reactions. Phosphorus promotes the accumulation of glucose phosphate and organic matter through phosphorylation. Heineman et al. (2016) found that LN and LP limit the accumulation of plant organic matter. The NPP of the grasslands is largely determined by LN and LP contents (Oldroyd and Leyser, 2020). Studies have shown that LN is closely related to plant growth and defense, and that the relationship between grassland NPP and LN and LP is affected by the relative distribution of these nutrients among plant tissues (Zheng et al., 2022). The higher resource utilization and competitiveness of plants are beneficial to improve NPP, however, plants in arid, water-deficient, and alpine environments usually have lower LN and higher LDMC (Yang et al., 2022). Therefore, even with an increase in LN and LP, NPP did not increase with it (Xu et al., 2018), which explains our results well. We also found that NPP of grassland ecosystems was positively correlated with LDMC (Forrestel et al., 2017). As LDMC increases, free water required for plant growth and development is gradually converted to bound water, which is involved in plant metabolic processes (e.g., photosynthesis). The NPP of DS decreased significantly with the increase of LA and SLA. Plants with small and thick leaves (smaller SLA) have better drought tolerance and were more conducive to cell material transport rate and photosynthetic efficiency (Poorter and Markesteijn, 2008; Osnas et al., 2018). These traits reflect the optimal utilization of limited resources by plants (Gupta et al., 2020). Although MAT of AM was significantly lower than DS (Supplementary Figure 2A), MAP (Supplementary Figure 2D), soil nitrogen content (Supplementary Figure 3A) and soil phosphorus content (Supplementary Figure 3B) were significantly higher than DS. This reflects that relatively abundant rainwater and soil nutrients can alleviate the impact of low-temperature stress on NPP (Berdugo et al., 2020).

Climatic factors are the main driving factors of spatial variation of grassland NPP (Wang et al., 2017a). We found that NPP of AM was significantly positively correlated with MAT and MCMT, and significantly negatively correlated with MAE. Numerous studies have shown that changes in NPP of AM are strongly correlated with MAT and MCMT (Keenan and Riley, 2018; Sloat et al., 2018; Shi et al., 2022). Temperature can not only directly affect the enzyme activities related to photosynthesis and respiration of plants, but also affects the production of organic compounds such as starch, protein, and lipid. Niu et al. (2019) found that global warming can improve the productivity of grassland in alpine regions for a long time. For NPP of AM, the influence of solar radiation is no less than that of temperature (Zheng et al., 2020), and the intensity of solar radiation is also one of the important factors affecting plant photosynthesis, which is the energy source of the grassland ecosystem (Brun et al., 2022). However, excessive evaporation triggered by too high radiation zone intensity can weaken the positive response of plants to high temperatures, causing plant water deficit and thus reducing NPP. Therefore, with the increase of MAE, the NPP of AM decreased significantly, and precipitation was the main climate factor leading to the temporal and spatial variation of the NPP of DS. Our conclusion also has been verified by a large number of previous studies (Liang et al., 2015; Erb et al., 2018; Guo et al., 2022). As the MAP increases, the NPP of AM increases significantly. Water restriction will seriously reduce the material transport efficiency and photosynthetic productivity efficiency of plants in arid areas, and reduce the organic matter yield of leaves and significantly affecting the productivity of the whole region (Sun et al., 2021). Significant difference analysis of climate factors of different grassland types found that the differences in climate factors (e.g., MAT, MWMT, MAP, and MAE; Supplementary Figure 2) and the differences in NPP (Figure 1B) showed surprising consistency, which also indirectly indicated that climate factors were the key environmental factors affecting grassland NPP (Yang et al., 2016).

Soil is a natural “storage box,” providing most of the nutrients needed for plants’ growth and reproduction. Soil nutrient elements are closely related to plant leaf nutrient elements (Gao et al., 2019). Soil nutrient factors were significantly positively correlated with community leaf traits such as LA, SLA, and LDMC (Huang et al., 2021). Climate affects the function of grassland ecosystems by changing the physicochemical properties of the soil. These results indicate that soil factors interact with other environmental factors to jointly affect grasslands NPP (Gong and Gao, 2019). With the increase of soil N and P, the NPP of AM decreased significantly. Soil N and P are essential nutrients for plant growth and development, which limit plant productivity (Gong H. et al., 2020). The alpine environment limits the decomposition capacity of soil microorganisms, and relatively low soil mineralization rate and soil nutrient availability inhibits the NPP of AM (Wang et al., 2017c). Nitrogen addition has been found to inhibit microbial respiration and thus affect the rate of microbial decomposition (Wang et al., 2018). Soil nutrient availability, the main soil factor, leading to change the NPP of AM (Blowes et al., 2019). Higher pH reduces soil nutrient availability by affecting soil organic matter storage (Chen et al., 2018). The availability of soil water and soil nutrients limit the NPP of DS. Precipitation can promote mineralization and improve soil nutrients, thereby increasing plant NPP, which is the limiting factor for plants to absorb soil nutrients. Jiang et al. (2017) also showed that NPP in arid areas is positively correlated with precipitation, and soil water can promote the decomposition of soil organic matter and thus improve the availability of soil nutrients. These findings explain the weak predictive power of soil N, soil P and soil pH for NPP of DS. Water shortage and strong evaporation lead to slow soil development and high soil pH, resulting in insufficient soil fertility and water (Zhao et al., 2022).

The NPP of AM (de = 62.4%) has stronger environment plasticity than DS (de = 43.5%). Regardless of AM or DS, the contribution of soil nutrients in shaping the NPP pattern was slightly higher than that of climate factors (Figure 5). Climatic factors, especially MAP and MAT, have long been considered key factors in grassland NPP (Erb et al., 2018). However, more and more studies have proved that soil properties play a dominant role in grassland NPP (Radujković et al., 2021). Temperature and precipitation control plant growth by changing microbial activity and soil physicochemical properties, thus affecting the NPP of grassland ecosystems (Newsham et al., 2016). Drought and frozen soil will lead to the decrease of soil mineralization rate and soil fertility (Nogueira et al., 2018). Soil is the habitat for plants, where providing water, inorganic matter, and organic matter for plant growth and development, and is one of the core elements linking the entire ecosystem. Climate change directly affects the soil environment and indirectly affects grassland NPP. Therefore, soil factors dominated the temporal and spatial changes of NPP in different grassland types on a large scale.

The wide study area and large amount of data in this paper ensure the credibility of our experimental results. Our study quantifies the effects of plant functional traits and environmental factors on NPP of AM and DS, providing important theoretical guidance for addressing global climate change and better management of grasslands. In future research, we will further study and explore more soil factors, such as soil microorganisms and soil N and P effectiveness on grassland NPP.

## Conclusion

We examined the relative roles of biological and abiotic factors in shaping NPP patterns using the data from 182 grassland plots in 17 DS and 21 AM in China. Our results confirmed that there are significant differences in productivity among different grasslands. Leaf traits, as well as climate and soil nutrient factors, jointly affect the changes of the grassland NPP. Among them, soil nutrients play the most critical role. Soil nutrient factors explained 61.8% of the spatial variation in NPP of AM and 44.4% of the spatial variation in NPP of DS. Quantifying the main driving factors of NPP of different grasslands is crucial for predicting future grassland dynamics and proposing reasonable management strategies.

## Data availability statement

The original contributions presented in this study are included in the article/Supplementary material, further inquiries can be directed to the corresponding author.

## Author contributions

JG: conceptualization, methodology, and investigation. XW, RW, and JG: formal analysis. XW: writing – original draft. All authors have read and agreed to the published version of the manuscript.

## Footnotes

1
https://data.cma.cn/

2
http://www.csdn.store

3
https://www.osgeo.cn/data/wc137

4
https://search.earthdata.NASA.gov/search

## Supplementary material

Supplementary Figure 1 Comparison of functional traits among different grassland types. **(A)** Variability between LA and the NPP of AM and DS; **(B)** variability between SLA and the NPP of AM and DS; **(C)** variability between LDMC and the NPP of AM and DS; **(D)** variability between LN and the NPP of AM and DS; **(E)** variability between LP and the NPP of AM and DS; **(F)** variability between N/P and the NPP of AM and DS. AM represents alpine meadows; DS represents deserts steppe; ns represents the insignificant difference; and *represents 0.01 < *P* < 0.05. SLA represents specific leaf area; LDMC represents leaf dry matter content; LN represents leaf nitrogen content; LP represents leaf phosphorus content; N/P represents leaf nitrogen and phosphorus ratio.

Supplementary Figure 2 Comparison of AM and DS with different climate factors. **(A)** Variability between MAT and the NPP of AM and DS; **(B)** variability between MCMT and the NPP of AM and DS; **(C)** variability between MWMT and the NPP of AM and DS; **(D)** variability between MAP and the NPP of AM and DS; **(E)** variability between ASD and the NPP of AM and DS; **(F)** variability between MAE and the NPP of AM and DS. AM represents alpine meadows; DS represents desert steppes; ns represents no significant difference between them; and ****represents *P* < 0.0001. MAT represents annual mean temperature; MCMT represents mean coldest monthly temperature; MWMT represents mean warmest monthly temperature; MAP represents mean annual precipitation; ASD represents annual sunshine hours; MAE represents average annual evaporation.

Supplementary Figure 3 Comparison of different soil nutrient factors (soil N, soil P, and soil PH) between AM and DS. **(A)** Variability between soil N and the NPP of AM and DS; **(B)** variability between soil P and the NPP of AM and DS; **(C)** variability between soil pH and the NPP of AM and DS. AM represents alpine meadows; DS represents desert steppes; ns represents the insignificant difference between the two; and ****represents *P* < 0.0001.
