# Supplementary material for: Precipitation and soil nutrients determine the spatial variability of grassland productivity at large scales in China
Source: Front Plant Sci. 2022 Sep 9;13:996313. doi: 10.3389/fpls.2022.996313 (PMC9505511; doi:10.3389/fpls.2022.996313)
Supplement: Supplementary Figure 1 — Comparison of functional traits among different grassland types. (A) Variability between LA and the NPP of AM and DS; (B) variability between SLA and the NPP of AM and DS; (C) variability between LDMC and the NPP of AM and DS; (D) variability between LN and the NPP of AM and DS; (E) variability between LP and the NPP of AM and DS; (F) variability between N/P and the NPP of AM and DS. AM represents alpine meadows; DS represents deserts steppe; ns represents the insignificant difference; and *represents 0.01 < P < 0.05. SLA represents specific leaf area; LDMC represents leaf dry matter content; LN represents leaf nitrogen content; LP represents leaf phosphorus content; N/P represents leaf nitrogen and phosphorus ratio. [file Data_Sheet_1.pdf]

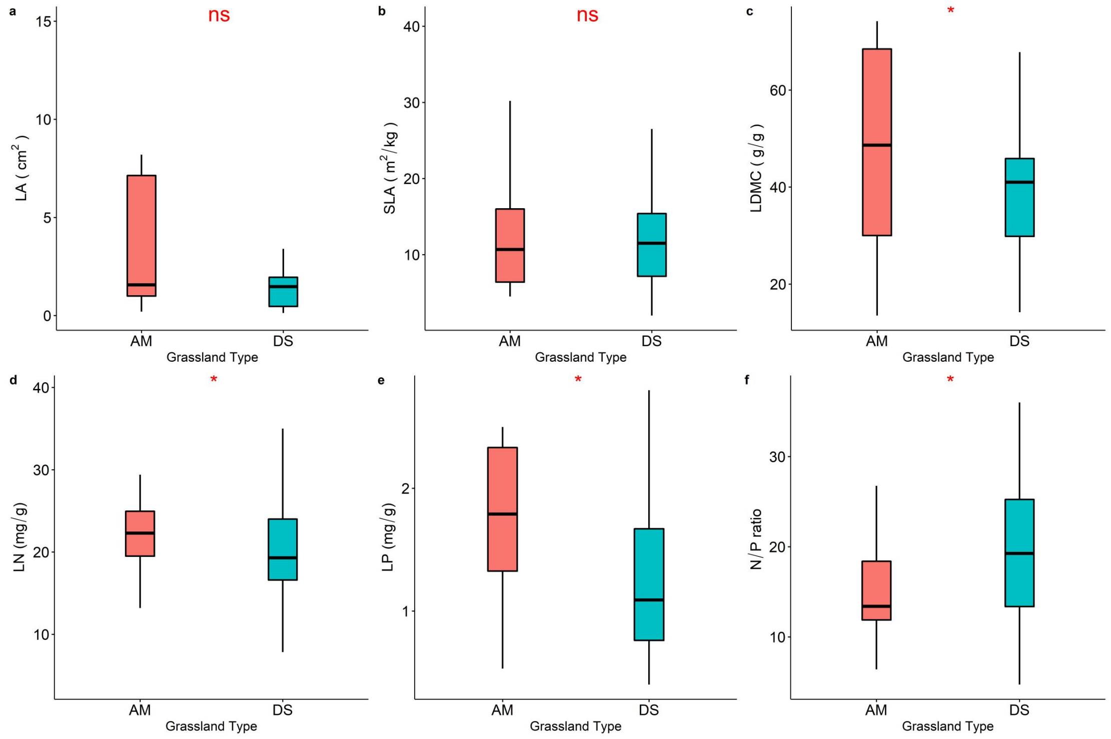

Fig. S1. Comparison of functional traits among different grassland types. a: variability between LA and the NPP of AM and DS; b: variability between SLA and the NPP of AM and DS; c: variability between LDMC and the NPP of AM and DS; d: variability between LN and the NPP of AM and DS; e: variability between LP and the NPP of AM and DS; f: variability between N/P and the NPP of AM and DS. AM represents alpine meadows; DS represents deserts steppe; ns represents the insignificant difference; and * represents $0.01<P<0.05$. SLA represents specific leaf area; LDMC represents leaf dry matter content; LN represents leaf nitrogen content; LP represents leaf phosphorus content; N/P represents leaf nitrogen and phosphorus ratio.

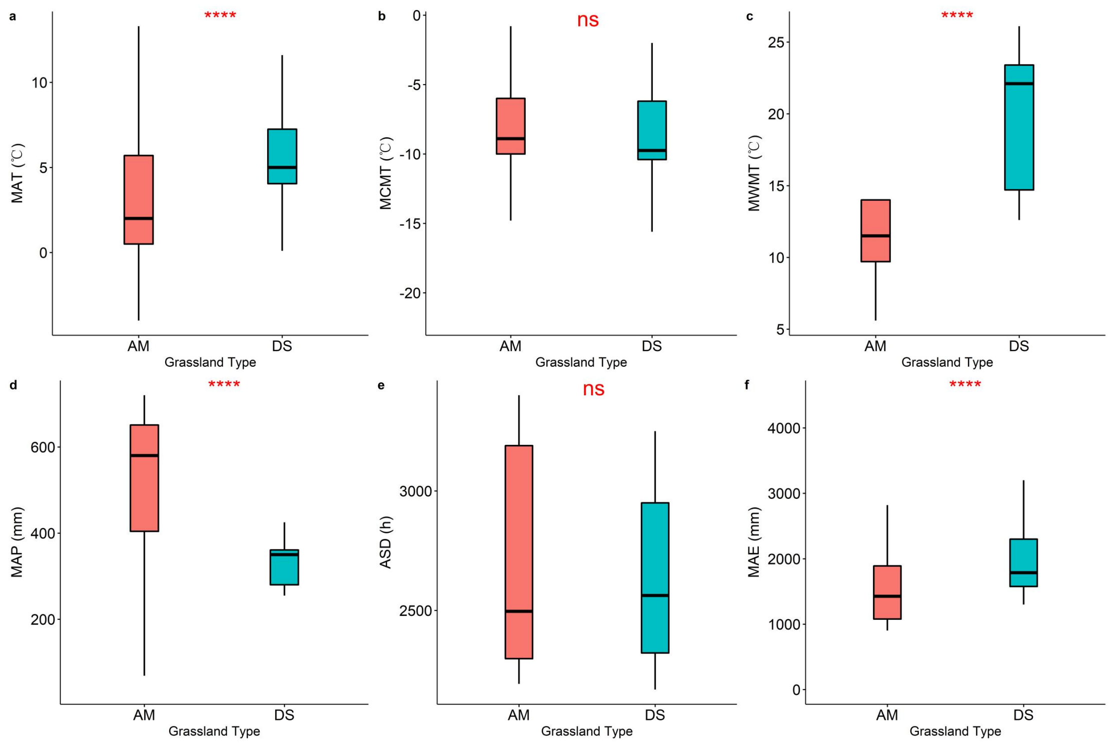

Fig. S2. Comparison of AM and DS with different climate factors. a: variability between MAT and the NPP of AM and DS; b: variability between MCMT and the NPP of AM and DS; c: variability between MWMT and the NPP of AM and DS; d: variability between MAP and the NPP of AM and DS; e: variability between ASD and the NPP of AM and DS; f: variability between MAE and the NPP of AM and DS. AM represents alpine meadows; DS represents desert steppes; ns represents no significant difference between them; and **** represents $P<0.0001$. MAT represents annual mean temperature; MCMT represents mean coldest monthly temperature; MWMT represents mean warmest monthly temperature; MAP represents mean annual precipitation; ASD represents annual sunshine hours; MAE represents average annual evaporation.

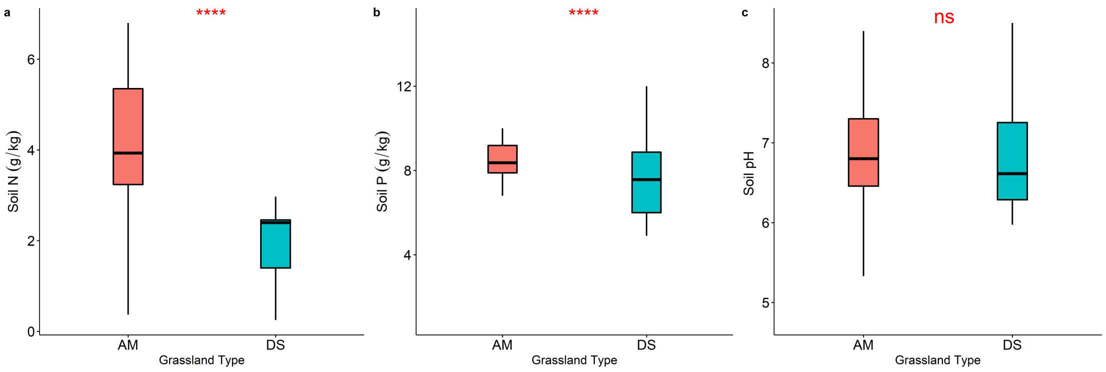

Fig. S3. Comparison of different soil nutrient factors (soil N, soil P, and soil PH) between AM and DS. a: variability between soil N and the NPP of AM and DS; b: variability between soil P and the NPP of AM and DS; c: variability between soil pH and the NPP of AM and DS. AM represents alpine meadows; DS represents desert steppes; ns represents the insignificant difference between the two; and **** represents P<0.0001.
